# Development of a New Radiofluorinated Quinoline Analog for PET Imaging of Phosphodiesterase 5 (PDE5) in Brain

**DOI:** 10.3390/ph9020022

**Published:** 2016-04-21

**Authors:** Jianrong Liu, Barbara Wenzel, Sladjana Dukic-Stefanovic, Rodrigo Teodoro, Friedrich-Alexander Ludwig, Winnie Deuther-Conrad, Susann Schröder, Jean-Michel Chezal, Emmanuel Moreau, Peter Brust, Aurélie Maisonial-Besset

**Affiliations:** 1 INSERM—Université d’Auvergne, UMR 990, IMTV, BP 184, F-63005 Clermont-Ferrand Cedex, France; liujianrongfr@gmail.com (J.L.); j-michel.chezal@udamail.fr (J.-M.C.); emmanuel.moreau@udamail.fr (E.M.); aurelie.maisonial@udamail.fr (A.M.-B.); 2 Helmholtz-Zentrum Dresden-Rossendorf, Institute of Radiopharmaceutical Cancer Research, Permoserstrasse 15, 04318 Leipzig, Germany; s.dukic-stefanovic@hzdr.de (S.D.-S.); r.teodoro@hzdr.de (R.T.); f.ludwig@hzdr.de (F.-A.L.); w.deuther-conrad@hzdr.de (W.D.-C.); s.schroeder@hzdr.de (S.S.); p.brust@hzdr.de (P.B.)

**Keywords:** PDE5, PET imaging, fluorine-18, quinoline, micellar chromatography

## Abstract

Phosphodiesterases (PDEs) are enzymes that play a major role in cell signalling by hydrolysing the secondary messengers cyclic adenosine monophosphate (cAMP) and/or cyclic guanosine monophosphate (cGMP) throughout the body and brain. Altered cyclic nucleotide-mediated signalling has been associated with a wide array of disorders, including neurodegenerative disorders. Recently, PDE5 has been shown to be involved in neurodegenerative disorders such as Alzheimer’s disease, but its precise role has not been elucidated yet. To visualize and quantify the expression of this enzyme in brain, we developed a radiotracer for specific PET imaging of PDE5. A quinoline-based lead compound has been structurally modified resulting in the fluoroethoxymethyl derivative **ICF24027** with high inhibitory activity towards PDE5 (IC_50_ = 1.86 nM). Radiolabelling with fluorine-18 was performed by a one-step nucleophilic substitution reaction using a tosylate precursor (RCY_(EOB)_ = 12.9% ± 1.8%; RCP > 99%; SA_(EOS)_ = 70–126 GBq/μmol). *In vitro* autoradiographic studies of [^18^F]**ICF24027** on different mouse tissue as well as on porcine brain slices demonstrated a moderate specific binding to PDE5. *In vivo* studies in mice revealed that [^18^F]**ICF24027** was metabolized under formation of brain penetrable radiometabolites making the radiotracer unsuitable for PET imaging of PDE5 in brain.

## 1. Introduction

The phosphodiesterases (PDEs) are a large family of enzymes that regulate the cellular levels of two important signalling molecules: the secondary messengers cyclic adenosine 3′,5′-monophosphate (cAMP) and cyclic guanosine 3′,5′-monophosphate (cGMP). This control results from their hydrolysing activity on the phosphodiester bonds of the free cyclic nucleotides. Consequently, PDEs regulate the cAMP and cGMP signalling pathways within the subcellular domains and play a critical role in various physiological and pathological processes, making them an attractive target for academic and industrial drug development studies [1,2]. To date, 21 genes encoding for PDEs have been identified and these enzymes are classified in 11 families (PDE1-11) depending on their amino acid sequence, substrate specificity, tissue distribution, regulatory and pharmacological properties. Most of the PDEs (PDE1, 2, 3, 10, and 11) are dual-substrate enzymes that hydrolyse both cAMP and cGMP but with different kinetic rates. PDE4, 7 and 8 are cAMP specific while PDE5, 6, and 9 only hydrolyse cGMP [3]. The PDE5 family comprises three isoforms in humans: PDE5A1, A2 and A3. It is well-known that PDE5 is localised in different organs and tissues such as platelets, lungs, and heart, for example [4], and is implicated in the regulation of vascular smooth muscle contraction and consequently of penile erection. For many years now, the pharmacological inhibition of smooth muscle contraction by PDE5 inhibitors has gained much interest for the treatment of erectile dysfunction [5]. To date only four PDE5 inhibitors have been approved by the Food and Drug Administration and by the European Medicines Agency e.g., vardenafil, tadalafil, avanafil and sildenafil. The latter (Viagra^®^) being the most representative molecule of this class of drugs.

Beside these peripheral physiological functions, PDE5 also plays an important role in the central nervous system. In the brain of mice and rats, expression of PDE5 has been demonstrated mainly in Purkinje cells of the cerebellum and in the hippocampus, both regions known to be associated with cognition processes [6,7,8,9,10]. In human brain tissue, beside the cerebellar distribution, PDE5 was also found in other regions such as substantia nigra, medulla, thalamus and the subthalamic nucleus as shown by PDE5 mRNA studies [11,12]. However, no quantitative data on the PDE5 expression in human brain are available yet. Nevertheless, the PDE5 enzyme has emerged as a potential therapeutic target for diseases related to neuroinflammatory and neurodegenerative processes because of its obvious relation to them [13]. Quite recently, a PCR analysis of *post-mortem* tissue from patients suffering from Alzheimer’s disease (AD) has discovered a considerable increase of PDE5 expression in the temporal cortex of brain compared with that of age-matched healthy controls [14]. Moreover, decreased levels of cGMP in the cerebrospinal fluid of patients with AD were observed which are assumed to be associated with the cognitive decline and amyloid pathology [14]. Given the lack of effective treatments for AD, PDE5 inhibitors as have been proposed as potential alternative cognitive enhancers [13,15]. The effectiveness of repeated PDE5 inhibitor treatment has been shown in several mouse models of AD and physiological aging [16,17]. Sildenafil, for example, prevented neuroinflammation, lowered beta-amyloid levels and improved cognitive performance in APP/PS1 transgenic mice [18]. Furthermore, chronic intraperitoneal injection of sildenafil downregulated the proapoptotic proteins caspase-3 and B-cell lymphoma 2-associated X and increased antiapoptotic molecules such as B-cell lymphoma protein-2 and brain-derived neurotrophic factor in aged mice [19]. All these findings highlight the need to improve the tools available to reassess both the *in vivo* effects of the different cGMP-PDE5 inhibitors and their role in pathological conditions. However, there is no validated method in the clinic to evaluate noninvasively the levels and distribution of PDE5 under normal or pathological conditions.

**Figure 1:**
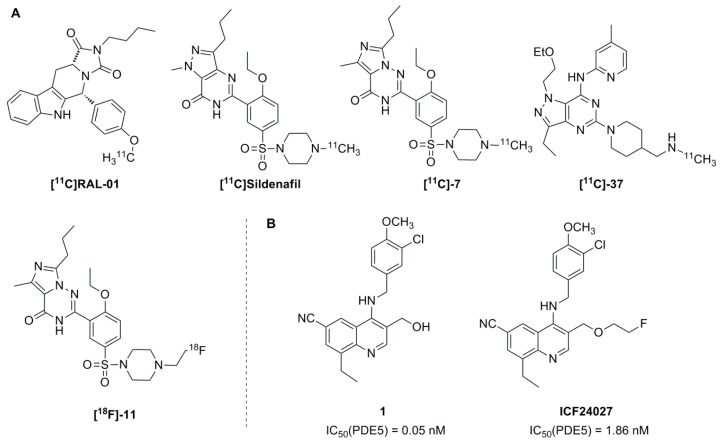
(**A**) Reported radiotracers for PET imaging of PDE5; (**B**) Selected lead compound **1** and novel derivative **ICF24027**.

Positron emission tomography (PET) is one of the most sensitive and unique functional imaging modalities among existing *in vivo* molecular imaging technologies through the use of radiotracers labelled with short-lived positron emitting radionuclides. To date only a few radiotracers have been investigated for PET imaging of intracellular pathways for signal transduction such as secondary messenger systems. Specific PET radiotracers of PDE5 would allow the quantification and localisation of PDE5 expression in the brain (availability, extent and intensity of up- or downregulation). Even if there are plenty specific and high affinity inhibitors of PDE5 already published, only a few radiotracers have been evaluated for PET imaging of this enzyme until now (Figure 1) [20,21,22]. However, because of their poor brain penetration or their lack of specific binding *in vivo* these radiotracers are not suitable for imaging of PDE5 in brain.

Moreover, most of them were labelled with carbon-11 (t_1/2_ = 20.4 min, 99% β^+^ emission, 960.5 keV) which is not the positron-emitting radionuclide of choice for developing radiotracers usable for PET imaging because of the relatively short half-live and low specific activities achievable. Fluorine-18 is more attractive for radiopharmaceutical chemistry and PET imaging, because of its well-adapted physical, chemical and nuclear characteristics (t_1/2_ = 109.8 min, 97% β^+^ emission, 633.5 keV). Thus, the development of radiofluorinated compounds with high affinity and selectivity towards PDE5 has to be extended and could provide an interesting alternative to the use of ^11^C-labelled radioligands.

With this view, we designed and synthesised a library of fluorinated compounds [23] based on the lead compound **1** (Figure 1) demonstrating a high affinity and selectivity towards PDE5 and the potential to cross the blood-brain barrier [24,25]. Out of this series of new compounds, the 2-fluoroethoxy derivative **ICF24027** has been identified as a potential candidate for ^18^F-labelling on the basis of its high *in vitro* inhibitory potency (Figure 1). In this report we describe the ^18^F-radiolabelling of **ICF24027** and its binding properties by *in vitro* autoradiography. Furthermore, the metabolism of [^18^F]**ICF24027** was investigated to evaluate its suitability as an *in vivo* imaging agent for PDE5 in brain.

## 2. Results

### 2.1. Organic Chemistry and Inhibitory Activity Towards PDEs

**Scheme 1:**
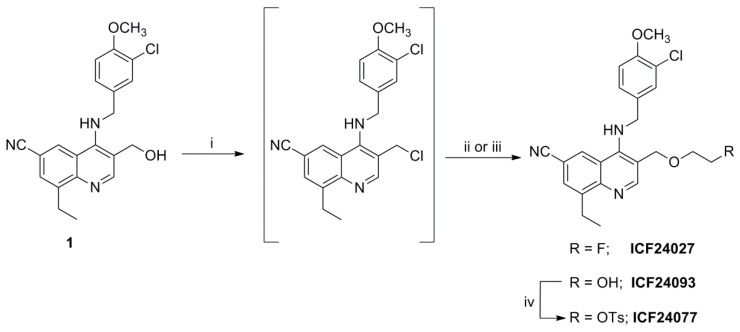
Synthetic pathway to the novel 2-fluoroethoxy derivative **ICF24027** and the tosylate precursor **ICF24077**. *Reagents and Conditions*: (i) SOCl_2_, 30 min, rt; (ii) 2-fluoroethanol, DMF, 80 °C, 30 h for **ICF24027**; (iii) ethylene glycol, DMF, 80 °C, 16 h for **ICF24093**; (iv) *p*-toluenesulfonyl chloride, NEt_3_, DMAP, CH_2_Cl_2_, rt, 20 h.

As depicted in Scheme 1, the synthesis of **ICF24027** was performed starting from alcohol **1** prepared according to the method previously described by Bi *et al.* [24]. Treatment with thionyl chloride afforded the corresponding chlorinated intermediate which reacted with an excess of 2-fluoroethanol in anhydrous *N*,*N*-dimethylformamide (DMF) to afford **ICF24027** in 29% yield. The corresponding tosylate precursor **ICF24077**, necessary for a one-step nucleophilic ^18^F-labelling, was synthesized via a two-step pathway which also involved the chlorinated intermediate (Scheme 1). Similarly to **ICF24027**, immediate reaction of the chlorinated intermediate with an excess of ethylene glycol in DMF gave the alcohol **ICF24093** in 73% yield. Reaction of **ICF24093** with *p*-toluenesulfonyl chloride in the presence of *N*,*N*-dimethylaminopyridine (DMAP) and triethylamine (NEt_3_) afforded the tosylate precursor **ICF24077** in 74% yield.

The inhibitory activity of **ICF24027** for the human recombinant PDE5A1 protein was evaluated by using an enzyme assay [26] with sildenafil as standard reference compound for comparison. With an IC_50_ value of 1.86 nM **ICF24027** showed a high inhibitory activity which is better than that of sildenafil (IC_50_ = 6.23 nM).

Moreover, the compound did not show valuable inhibition against PDE2A3, PDE3A, PDE4A1 and C2, PDE6AB, PDE9A1, PDE10A1, and PDE11A1 [23]. The substitution of the OH group by a 2-fluoroethyl functionality may have resulted in a decrease of the inhibitory activity of **ICF24027** compared to the lead compound **1** with a published IC_50_ value of 0.05 nM [24]. However, a direct comparison of the two IC_50_ values is difficult because different enzyme assays were used, probably resulting in variations independent from the structure of the molecule. 

### 2.2. Radiochemistry

**Scheme 2:**
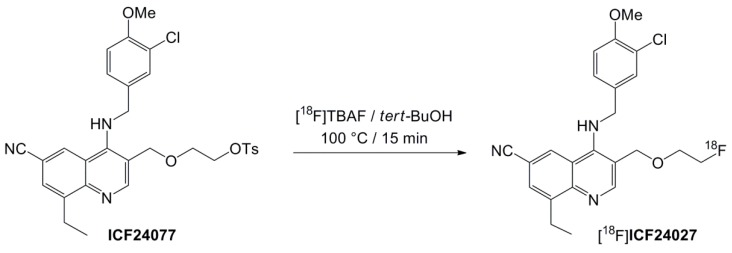
^18^F-Radiolabelling of [^18^F]**ICF24027**.

The new radioligand [^18^F]**ICF24027** was prepared by nucleophilic substitution of the tosylate precursor **ICF24077** using anhydrous [^18^F]tetra-*n*-butyl ammonium fluoride ([^18^F]TBAF) in *tert*-BuOH (Scheme 2). After 15 min reaction time under conventional heating at 100 °C, no considerable increase of labelled product was observed, resulting in labelling efficiencies of 41.8% ± 5.1% (*n* = 6). Beside [^18^F]fluoride, two radioactive by-products (<10% of total radioactivity) were observed according to radio-TLC analysis. Interestingly, when the classical K[^18^F]F-K_2.2.2_-carbonate complex system and acetonitrile (ACN) at 90 °C or DMSO at 120 °C were used, considerably lower labelling yields of <10% were achieved. The precursor remained quite stable under all conditions tested as proven by HPLC.

**Figure 2:**
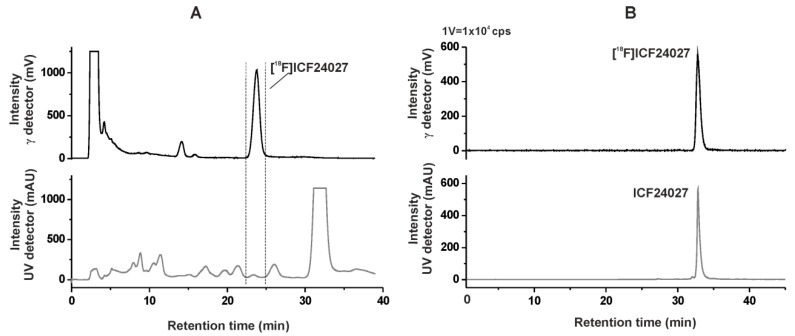
(**A**) Semi-preparative radio- and UV-HPLC chromatograms of [^18^F]**ICF24027** (conditions: Reprosil-Pur C18-AQ, 250 mm × 10 mm, 62% ACN/20 mM NH_4_OAc_aq._, 3.0 mL/min). (**B**) Analytical radio- and UV-HPLC chromatograms of the final product of [^18^F]**ICF24027** spiked with the nonradioactive reference **ICF24027** (conditions: Reprosil-Pur C18-AQ, 250 mm × 4.6 mm, gradient with eluent mixture of ACN/20 mM NH_4_OAc aq., 1.0 mL/min).

The isolation of [^18^F]**ICF24027** was performed by using semi-preparative RP-HPLC. The product was collected at a retention time of 22–25 min (Figure 2A), purified using solid phase extraction on an RP cartridge and formulated in sterile isotonic saline containing 10% of EtOH for better solubility. Analytical radio- and UV-HPLC of the final product, spiked with the nonlabelled reference compound, confirmed the identity of [^18^F]**ICF24027** (Figure 2B). Finally, the radiotracer was obtained with a radiochemical purity of ≥99%, in radiochemical yields (EOB) of 12.9% ± 1.8% (*n* = 6, decay corrected), and specific activities (EOS) in the range of 70–126 GBq/μmol using starting activities of 2–3 GBq.

The stability of the radiotracer was investigated by incubation at 40 °C in (i) phosphate-buffered saline (PBS) and (ii) pig plasma samples. [^18^F]**ICF24027** proved to be stable in these media, and no radiodefluorination or degradation was observed within 60 min of incubation time.

### 2.3. In Vitro Autoradiographic Studies

**Figure 3:**
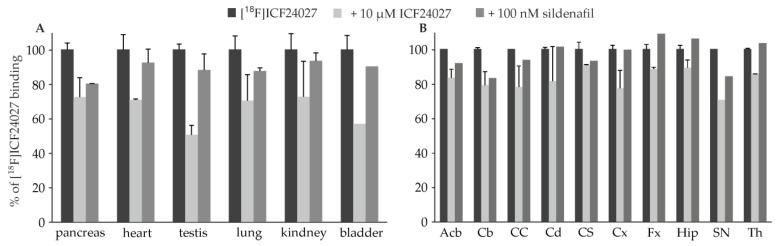
Selectivity of the binding of [^18^F]**ICF24027** in (**A**) different mouse tissue and (**B**) porcine brain *in vitro* (*n* = 2). Remaining binding of 5.26 nM [^18^F]**ICF24027** in the presence of 10 μM **ICF24027** or 100 nM sildenafil is presented as percentage of total binding (=100%). Abbreviations: Acb = Nucleus accumbens, Cb = Cerebellum, CC = Corpus callosum, Cd = Nucleus caudatus, Cx = Cortex, CS = Colliculus superior, Fx = Fornix, Hip = Hippocampus, SN = Substantia nigra, Th = Thalamus.

Guided by the work of Lakics *et al.* [11] we investigated [^18^F]**ICF24027** in mouse tissue characterised with high PDE5 expression using autoradiography. The highest binding was detected in heart and pancreas followed by kidney, lung, testis and bladder which is in accordance to reported expression data [7,11]. The competitive binding studies with **ICF24027** and sildenafil showed high specific PDE5 binding in pancreas, whereas in heart, testis and bladder sildenafil seems to compete moderately with binding sites of [^18^F]**ICF24027** (Figure 3A).

Sildenafil does not cross-react significantly with any PDE other than PDE6, which is present mainly in retina [27]. Hence, the difference in competitive binding of **ICF24027** and sildenafil indicates that [^18^F]**ICF24027** probably binds to another target. Our assumption that σ_1_ or σ_2_ receptors could be involved has been proven wrong, since the *K*_i_ values of **ICF24027** determined for both, σ_1_ and σ_2_, are higher than 10^−6^ M.

**Figure 4:**
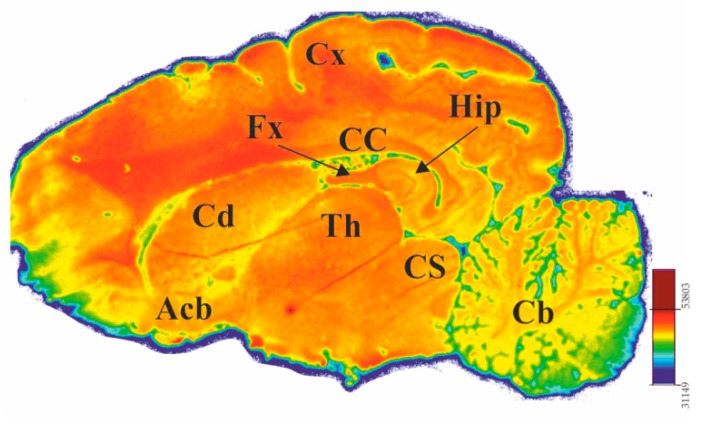
Representative sagittal porcine brain slice showing distribution of the binding sites of [^18^F]**ICF24027** *in vitro* (total binding at 5.26 nM). Abbreviations: Acb = Nucleus accumbens, Cb = Cerebellum, CC = Corpus callosum, Cd = Nucleus caudatus, Cx = Cortex, CS = Colliculus superior stratum, Fx = Fornix, Hip = Hippocampus, Th = Thalamus.

To investigate the distribution of binding sites of [^18^F]**ICF24027** in brain, we performed *in vitro* autoradiographic studies on porcine brain slices. The most dense binding was detected in the substantia nigra (SN), followed by corpus callosum (CC) which is in accordance with the findings of Loughney *et al.* for human brain tissue [12]. However, a modest level of binding of [^18^F]**ICF24027** was observed in the cerebellum (Cb) and hippocampus (Hip) which are also described to express PDE5 mRNA in higher levels (Figure 4). Competition with 10 μM **ICF24027** showed a slight decrease of [^18^F]**ICF24027** binding, from 10% in colliculus superior (CS) to 30% in SN, suggesting a high nonspecific binding of the radioligand (Figure 3B). Furthermore, a reduction of binding of [^18^F]**ICF24027** in the range of 20% was observed in Cb and SN in the presence of sildenafil. In addition, the modest specific binding of [^18^F]**ICF24027** could be due to an estimated low expression of PDE5 in the brain. Based on a compilation of biochemical data, Cumming [28] postulated recently that the density is in nanomolar range, hence, a ligand with subnanomolar affinity would be necessary for quantification of PDE5 in brain.

### 2.4. Metabolism Studies of [^18^F]**ICF24027** In Vivo and In Vitro

*In vivo* metabolism of [^18^F]**ICF24027** was investigated in plasma and brain samples obtained from CD-1 mice (*n* = 2) at 30 min post injection of 55 MBq of the radioligand. Analyses of the samples were performed by using two different methods: (A) direct injection of the samples into a micellar chromatography system (MLC) and (B) injection of the samples into a RP-HPLC system by using extracts obtained after protein precipitation. MLC allows one to analyse biological samples without eliminating the tissue matrix due to the ability of micellar aggregates to dissolve the proteins and other components. Beside a reduction of analysis time, this method also ensures a quantitative analysis, which is often not given when proteins are precipitated and bound polar or ionic metabolites are not entirely extractable. The MLC method was recently investigated by Nakao *et al.* regarding its suitability for plasma metabolite analysis of PET radioligands in clinical use [29]. Our group extended these experiments by using this method to analyse homogenates of brain samples [30]. Most commonly sodium dodecyl sulphate (SDS) as anionic surfactant and alcohols or ACN as organic modifiers are used for MLC. Therefore, as eluent a mixture of an aqueous solution of SDS, phosphate buffer and propan-1-ol was used in a gradient mode starting under micellar conditions (3% propan-1-ol) to elute the protein fraction completely accompanied by the release of protein bound ^18^F-compounds. Subsequent increase of the amount of propan-1-ol as organic modifier leads to high submicellar conditions [29,31,32] resulting in the elution of the intact radioligand. To additionally analyse the samples by RP-HPLC, plasma and brain homogenates were treated with a mixture of ice-cold ACN/water to precipitate the proteins resulting in a nearly quantitative recovery of extracted radioactivity of >97%.

**Figure 5:**
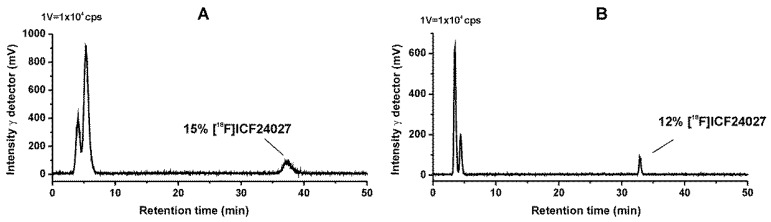
Representative radio chromatograms of a mouse brain sample of [^18^F]**ICF24027** 30 min p.i. (**A**) Micellar chromatography (conditions: Reprosil-Pur C18-AQ, 250 mm × 4.6 mm, gradient: 3%–30%–3% 1-PrOH/100 mM SDS aq., 10 mM Na_2_HPO_4_ aq.; flow: 0.75 mL/min); (**B**) Reversed phase chromatography (conditions: Reprosil-Pur C18-AQ, 250 mm × 4.6 mm, gradient: 10%–90%–10% ACN/20 mM NH_4_OAc aq.; flow 1.0 mL/min).

With both methods a high fraction of radiometabolites was detected in plasma with only 7% of total radioactivity representing the non-metabolized [^18^F]**ICF24027**. Two radioactive metabolites were observed, which we assume to be very polar based on their short retention times in both MLC and RP-HPLC chromatograms. Moreover, these two radiometabolites were also observed in brain samples indicating their ability to cross the blood-brain barrier. Thus, only 12%–15% of total activity were represented by intact [^18^F]**ICF24027** in brain (Figure 5).

For comparison we performed metabolism studies *in vitro* with both the radioactive [^18^F]**ICF24027** and the nonradioactive compound **ICF24027** by using mouse liver microsomes in the presence of NADPH. After 60 min incubation of [^18^F]**ICF24027**, no intact tracer and a slightly different radio-MLC and -HPLC profile was observed compared to the *in vivo* data. Mainly a single polar radiometabolite accounting 93% of the total activity was detected (data not shown). As observed also for other radiotracers investigated in our group, the metabolism may be faster *in vitro*, depending on the assay conditions and therefore probably resulting in a ^18^F-containing product originating from metabolic intermediates after further oxidation processes. Investigation of the non-radioactive compound *in vitro* demonstrated three major metabolites in the UV-HPLC chromatogram. One of them, with a retention time of 29 min, could be identified as the alcohol **1**.

## 3. Discussion

The objective of this work was the development of a specific radiotracer for imaging of PDE5 in brain. We designed and successfully synthesised the fluorinated quinoline derivative **ICF24027** which demonstrated high inhibitory potency towards PDE5 and excellent specificity towards other PDEs in particular PDE6 and PDE11 as determined by a cAMP/cGMP-based enzyme assay. The corresponding ^18^F-labelled radiotracer [^18^F]**ICF24027** was obtained with a high radiochemical purity (≥99%) and good specific activities in the range of 70–126 GBq/μmol. *In vitro* autoradiographic studies on porcine brain slices and different mouse tissue revealed specific binding of [^18^F]**ICF24027** in certain brain regions and in pancreas. However, a high non-specific binding of [^18^F]**ICF24027** was also observed, probably resulting from the high lipophilicity of the compound (log*D* of 3.9 calculated using ACD/Labs).

*In vivo* metabolism studies in mice revealed a fast metabolism of [^18^F]**ICF24027** with the formation of brain penetrable radiometabolites. Thus, only 12%–15% of total activity in the brain were represented by intact [^18^F]**ICF24027** after 30 min p.i. making this radiotracer unsuitable for specific imaging of PDE5 in brain. We assume that the polar radiometabolites are generated by *O*-dealkylation of [^18^F]**ICF24027** that takes place on the basis of cytochrome P450-catalyzed hydroxylations at sp^3^-C-atoms bearing an N-, O-, or S-heteroatom [33,34]. The resulting polar radiometabolites are [^18^F]fluoroethanol and its oxidation products [^18^F]fluoroacetaldehyde and [^18^F]fluoroacetate, which are known to cross the blood-brain barrier [35]. The nonradioactive counterpart of a *O*-dealkylation of [^18^F]**ICF24027** is identical to the alcohol **1** and was detected in the corresponding *in vitro* studies. Radiodefluorination is expected to play a minor role according to the meanwhile accepted assumption of trapping the [^18^F]fluoroacetate as [^18^F]fluoroacetyl-CoA inside the cells [36]. Moreover, it is assumed that the elimination of [^18^F]fluoride is caused by oxidation of the carbon atom next to the fluorine. For the [^18^F]fluoroethyl group this fluorine is in β-position to the heteroatom and is found to be metabolically cleaved at a slow rate because of the β-heteroatom effect [37,38]. This is in contrast to the longer-chain [^18^F]fluoropropyl group which demonstrated strong radiodefluorination as recently described for different ^18^F-labelled PET-tracers [34,37,39].

The formation of brain penetrable radiometabolites on the basis of *O*-dealkylation was already observed for a PDE2 ligand recently developed in our group [30] and for other ligands bearing fluoroalkoxy side chains bound on an aromatic ring [40,41]. By contrast, also several comparable radiotracers are described which resist to this specific metabolism over a certain time span which would be sufficient for a PET scan [42,43]. Obviously, this metabolic pathway depends considerably on the whole structure of a molecule and its binding potential towards the cytochrome P450 enzyme. Therefore, a prediction regarding the metabolic stability seems to be rather difficult.

One strategy to increase the stability could be deuteration in the direct neighbourhood of the ^18^F-fluorine by a deuterium-proton exchange at the corresponding carbon atom [34]. However, fluorination on another moiety of the molecule and keeping the OH group unsubstituted as in the lead compound **1**, could lead to an improvement of the inhibitory activity combined with a decreased lipophilicity. The latter is known to be an important criterion regarding blood-brain barrier permeability and probably caused the high nonspecific binding in our *in vitro* autoradiographic experiments.

Finally, the results of the study presented in this report will be useful for the design of metabolically more stable fluorinated quinoline analogs for further development of PET probes for imaging of PDE5 in brain.

## 4. Materials and Methods

### 4.1. Organic Chemistry

#### 4.1.1. General Information

All commercially available reagents and solvents were purchased at the following commercial suppliers: Sigma Aldrich (Saint-Quentin Fallavier, France), Acros Organics (Geel, Belgium), Fisher Scientific (Illkirch, France), Carlo Erba Reagents (Val de Reuil, France), VWR (Fontenay-sous-Bois, France) and Alfa Aesar (Karlsruhe, Germany) and were used without further purification. Room temperature (rt) refers to 20–25 °C. All solvents were dried using common techniques [44]. Air and moisture sensitive reactions were carried out under dry argon atmosphere. Reaction progress was monitored by thin layer chromatography (TLC) performed using silica 60 F254 or alumina gel 60A + F254 plates and visualized with UV light (UV lamp Fisher Scientific, 365 nm or 254 nm). Column chromatography was performed on silica gel (Chromagel 60 ACC, 40–63 μm, Carlo Erba Reagents). Uncorrected melting points (mp) were measured on an IA9100 Digital Melting Point Apparatus (Bibby Scientific, Roissy, France). Infrared spectra (IR) were recorded in the range 4000–440 cm^−1^ on a IS10 with attenuated total reflectance (ATR) accessory Nicolet (Fisher Scientific). Nuclear magnetic resonance (NMR) spectra were recorded on an Avance 500 instrument (500 MHz for ^1^H, 125 MHz for ^13^C) or an Avance 400 instrument (400 MHz for ^1^H, 100 MHz for ^13^C) (Bruker Biospin SAS, Wissembourg, France). All chemical shifts (δ) are reported in parts per million (ppm). To describe spin multiplicity, standard abbreviations such as s, d, t, q, m, brs, dd referring to singlet, doublet, triplet, quartet, multiplet, broad singlet and doublet of doublet respectively, are used. The coupling constants (*J*) are given in Hz. All new compounds were analysed by High-Resolution Mass Spectrometry (HRMS, Waters^®^ Micromass^®^ Q-Tof micro™ Mass Spectrometer, UBP-START, Blaise Pascal University, Clermont-Ferrand, France).

#### 4.1.2. Syntheses

##### 4-[(3-Chloro-4-methoxybenzyl)amino]-8-ethyl-3-[(2-fluoroethoxy)methyl]quinoline-6-carbonitrile *(**ICF24027**)*

A solution of alcohol **1** [24] (250 mg, 0.65 mmol) was stirred in thionyl chloride (4 mL) at rt and under argon atmosphere for 30 min. Then the solvent was evaporated under reduced pressure and the yellow residue obtained was stored under vacuum in a desiccator overnight. The product was dissolved in dry DMF (15 mL) and 2-fluoroethanol (0.38 mL, 6.54 mmol, 10.0 eq.) was added at rt under stirring and argon atmosphere. The resulting mixture was stirred at 80 °C for 30 h. After cooling to rt, a saturated aqueous sodium hydrogen carbonate solution (30 mL) was added. The mixture was extracted with ethyl acetate (3 × 30 mL). The combined organic layers were washed with brine (2 × 40 mL), dried over anhydrous magnesium sulphate, filtered and then concentrated under reduced pressure. The crude product was purified by column chromatography (SiO_2_, ethyl acetate/cyclohexane, 2/8 to 5/5, *v*/*v*) to give **ICF24027** (80 mg, 0.19 mmol) as a light yellow solid. Yield: 29%; mp: 150 ± 1 °C; IR (ATR accessory) ν: 3390, 2927, 2800, 2229, 1505, 1260, 1104 cm**^−1^**; ^1^H-NMR (CDCl_3_, 400 MHz) δ 8.53 (s, 1H, H_ar_), 8.32 (d, 1H, ^4^*J* = 1.6 Hz, H_ar_), 7.59 (m, 1H, H_ar_), 7.34 (d, 1H, ^4^*J* = 2.2 Hz, H_ar_), 7.17 (dd, 1H, ^3^*J* = 8.4 Hz , ^4^*J* = 2.2 Hz, H_ar_), 6.91 (d, 1H, ^3^*J* = 8.4 Hz, H_ar_), 5.78 (brs, 1H, NH), 4.71 (d, 2H, ^3^*J* = 5.1 Hz, CH_2_NH), 4.62 (s, 2H, CH_2_OCH_2_CH_2_F), 4.52 (m, 2H, CH_2_OCH_2_CH_2_F), 3.89 (s, 3H, OCH_3_), 3.68 (m, 2H, CH_2_OCH_2_CH_2_F), 3.22 (q, 2H, ^3^*J* = 7.5 Hz, CH_2_CH_3_), 1.33 (t, 3H, ^3^*J* = 7.5 Hz, CH_2_CH_3_); ^13^C-NMR (CDCl_3_, 100 MHz) δ 154.8 (C_ar_OCH_3_), 153.1 (C_ar_NH), 152.5 (CH_ar_), 149.8 (C_ar_), 145.0 (C_ar_CH_2_CH_3_), 132.0 (C_ar_), 129.3 (CH_ar_), 128.2 (CH_ar_), 128.1 (CH_ar_), 126.8 (CH_ar_), 123.0 (C_ar_Cl), 119.9 (C_ar_), 119.5 (CN), 114.8 (C_ar_CH_2_O), 112.4 (CH_ar_), 107.5 (C_ar_CN), 82.7 (d, ^1^*J*_C-F_ = 169 Hz, OCH_2_CH_2_F), 70.1 (CH_2_OCH_2_CH_2_F), 69.0 (d, ^2^*J*_C-F_ = 19 Hz, OCH_2_CH_2_F), 56.2 (OCH_3_), 52.0 (CH_2_NH), 24.9 (CH_2_CH_3_), 14.4 (CH_2_CH_3_); HRMS: *m*/*z* 428.1429 [M + H]^+^ (counted for [C_23_H_24_ClFN_3_O_2_]^+^ 428.1541).

##### 4-[(3-Chloro-4-methoxybenzyl)amino]-8-ethyl-3-[(2-hydroxyethoxy)methyl]quinoline-6-carbonitrile *(**ICF24093**)*

A solution of **1** [24] (0.10 g, 0.26 mmol) was stirred in thionyl chloride (5 mL) under argon atmosphere at rt for 30 min. The solvent was removed under reduced pressure and the residue obtained was dissolved in dry DMF (10 mL) under argon atmosphere. Then, ethylene glycol (0.16 g, 2.61 mmol, 10 eq.) was added at rt and the mixture was stirred at 80 °C for 16 h. After cooling to rt, a saturated sodium hydrogen carbonate solution (20 mL) was added dropwise and the resulting mixture was extracted with ethyl acetate (3 × 25 mL). The combined organic layers were washed with brine (3 × 50 mL), dried over anhydrous magnesium sulphate, filtered, and then concentrated under reduced pressure. The crude product was purified by column chromatography (SiO_2_, ethyl acetate/ethanol, 9/1, *v*/*v*) to yield ICF24093 (80 mg, 0.19 mmol) as a white solid. Yield: 73%; mp: 141 ± 1 °C; IR (ATR accessory) ν: 3388, 2932, 2874, 2229, 1504, 1259 cm^−1^; ^1^H-NMR (CDCl_3_, 400 MHz) δ 8.54 (s, 1H, H_ar_), 8.30 (d, 1H, ^4^*J* = 1.8 Hz, H_ar_), 7.60 (m, 1H, H_ar_), 7.35 (d, 1H, ^4^*J* = 2.2 Hz, H_ar_), 7.16 (dd, 1H, ^3^*J* = 8.4 Hz , ^4^*J* = 2.2 Hz, H_ar_), 6.91 (d, 1H, ^3^*J* = 8.4 Hz, H_ar_), 5.83 (brs, 1H, NH), 4.70 (d, 2H, ^3^*J* = 5.4 Hz, CH_2_NH), 4.61 (s, 2H, CH_2_OCH_2_CH_2_OH), 3.90 (s, 3H, OCH_3_), 3.73 (m, 2H, OCH_2_CH_2_OH), 3.56 (m, 2H, OCH_2_CH_2_OH), 3.23 (q, 2H, ^3^*J* = 7.4 Hz, CH_2_CH_3_), 1.35 (t, 3H, ^3^*J* = 7.4 Hz, CH_2_CH_3_); ^13^C-NMR (CDCl_3_, 125 MHz) δ 154.9 (C_ar_OCH_3_), 153.6 (C_ar_NH), 151.7 (CH_ar_), 149.8 (C_ar_), 144.4 (C_ar_CH_2_CH_3_), 131.9 (C_ar_), 129.4 (CH_ar_), 128.6 (CH_ar_), 128.5 (CH_ar_), 126.9 (CH_ar_), 123.2 (C_ar_Cl), 119.7 (C_ar_), 119.4 (CN), 115.2 (C_ar_CH_2_O), 112.5 (CH_ar_), 107.6 (C_ar_CN), 71.4 (OCH_2_CH_2_OH), 69.9 (CH_2_OCH_2_CH_2_OH), 61.8 (OCH_2_CH_2_OH), 56.4 (OCH_3_), 52.1 (CH_2_NH), 25.0 (CH_2_CH_3_), 14.4 (CH_2_CH_3_). HRMS: *m*/*z* 426.1604 [M + H]^+^ (counted for [C_23_H_25_ClN_3_O_3_]^+^ 426.1584).

##### 2-((4-[(3-Chloro-4-methoxybenzyl)amino]-6-cyano-8-ethylquinolin-3-yl)methoxy)ethyl 4-methylbenzene-sulfonate *(**ICF24077**)*

To a solution of ICF24093 (80 mg, 0.19 mmol) in dry dichloromethane (15 mL) were successively added under stirring and argon atmosphere, *p*-toluenesulfonyl chloride (0.11 g, 0.57 mmol, 3.0 eq.), triethylamine (0.10 mL, 0.75 mmol, 4.0 eq.) and DMAP (9.1 mg, 74.5 μmol, 0.4 eq.). The resulting mixture was stirred at rt for 20 h before addition of water (20 mL). After decantation, the aqueous layer was extracted with dichloromethane (3 × 20 mL). The combined organic layers were washed with brine (50 mL), dried over sodium sulphate, filtered and concentrated under reduced pressure. The crude product was purified by column chromatography (SiO_2_, ethyl acetate) to obtain ICF24077 (80 mg, 0.14 mmol) as a white solid. Yield: 74%; mp: 140 ± 1 °C; IR (ATR accessory) ν: 3403, 2965, 2223, 1502, 1346, 1256, 1169 cm^−1^; ^1^H-NMR (CDCl_3_, 500 MHz) δ 8.47 (s, 1H, H_ar_), 8.33 (s, 1H, H_ar_), 7.70 (d, 2H, ^3^*J* = 8.2 Hz, 2H_ar_), 7.60 (s, 1H, H_ar_), 7.31 (d, 1H, ^4^*J* = 1.3 Hz, H_ar_), 7.25 (d, 2H, ^3^*J* = 8.2 Hz, 2H_ar_), 7.18 (dd, 1H, ^3^*J* = 8.3 Hz, ^4^*J* = 1.3 Hz, H_ar_), 6.92 (d, 1H, ^3^*J* = 8.3 Hz, H_ar_), 5.92 (brs, 1H, NH), 4.72 (d, 2H, ^3^*J* = 5.8 Hz, CH_2_NH), 4.53 (s, 2H, CH_2_OCH_2_CH_2_OH), 4.14 (m, 2H, OCH_2_CH_2_OTs), 3.88 (s, 3H, OCH_3_), 3.63 (m, 2H, OCH_2_CH_2_OTs), 3.22 (q, 2H, ^3^*J* = 7.4 Hz, CH_2_CH_3_), 2.38 (s, 3H, CH_3_C_ar_), 1.36 (t, 3H, ^3^*J* = 7.4 Hz, CH_2_CH_3_). ^13^C-NMR (CDCl_3_, 125 MHz) δ 154.6 (C_ar_OCH_3_), 152.8 (C_ar_NH), 152.8 (CH_ar_), 149.8 (C_ar_), 145.1 (C_ar_CH_3_), 144.9 (C_ar_CH_2_CH_3_), 132.6 (C_ar_), 132.2 (C_ar_), 129.9 (2CH_ar_), 129.1 (CH_ar_), 128.1 (2CH_ar_), 127.8 (2CH_ar_), 126.8 (CH_ar_), 122.7 (C_ar_Cl), 119.9 (C_ar_), 119.5 (CN), 114.3 (C_ar_CH_2_O), 112.4 (CH_ar_), 107.4 (C_ar_CN), 70.0 (CH_2_OCH_2_CH_2_OTs), 68.9 (OCH_2_CH_2_OTs), 67.4 (OCH_2_CH_2_OTs), 56.2 (OCH_3_), 51.5 (CH_2_NH), 24.8 (CH_2_CH_3_), 21.6 (C_ar_CH_3_), 14.4 (CH_2_CH_3_). HRMS: *m*/*z* 580.1674 [M + H]^+^ (counted for [C_30_H_31_ClN_3_O_5_S]^+^ 580.1673).

### 4.2. Radiochemistry

#### 4.2.1. General

No-carrier-added [^18^F]fluoride was produced via the [^18^O(p,n)^18^F] nuclear reaction by irradiation of an [^18^O]H_2_O target (Hyox 18 enriched water, Rotem Industries Ltd, Mishor Yamin, Israel) on a Cyclone 18/9 (iba RadioPharma Solutions, Louvain-La-Neuve, Belgium) with fixed energy proton beam using Nirta [^18^F]fluoride XL target.

Radio thin layer chromatography (radio-TLC) was performed on silica gel (Polygram^®^ SIL G/UV_254_) pre-coated plates with a mixture of EtOAc/cyclohexane 3/1 (*v*/*v*) as eluent. The plates were exposed to storage phosphor screens (BAS-TR2025, Fujifilm Co., Tokyo, Japan) and recorded using a bio-imaging analyser system (BAS-1800 II, Fujifilm). Images were evaluated with the BASReader and AIDA 2.31 software (raytest Isotopenmessgeräte GmbH, Straubenhardt, Germany).

Analytical chromatographic separations were performed on a LC-2000 system (JASCO, Groß-Umstadt, Germany) incorporating a PU-2080*Plus* pump, AS-2055*Plus* auto injector (100 μL sample loop), and a UV-2070*Plus* detector coupled with a gamma radioactivity HPLC detector (Gabi Star, raytest Isotopenmessgeräte GmbH, Straubenhardt, Germany). Data analysis was performed with the Galaxie chromatography software (Agilent Technologies, Santa Clara, CA, USA) using the chromatograms obtained at 254 nm. A Reprosil-Pur C18-AQ column (250 mm × 4.6 mm; 5 μm; Dr. Maisch HPLC GmbH; Ammerbuch-Entringen, Germany) with ACN/20 mM NH_4_OAc aq. (pH 6.8) as eluent mixture and a flow of 1.0 mL/min was used (gradient: eluent A 10% ACN/20 mM NH_4_OAc aq.; eluent B 90% ACN/20 mM NH_4_OAc aq.; 0–10 min 100% A, 10–30 min up to 100% B, 30–40 min 100% B , 40–50 min up to 100% A, 50–60 min 100% A).

Semi-preparative HPLC separations were performed on a JASCO LC-2000 system, incorporating a PU-2080-20 pump, an UV/VIS-2075 detector coupled with a gamma radioactivity HPLC detector whose measurement geometry was slightly modified (Gabi Star, raytest Isotopenmessgeräte GmbH) and a fraction collector (CHF-122SC, Advantec, Dublin, CA, USA). A Reprosil-Gold 120 C18 column (250 mm × 10 mm; 10 μm; Dr. Maisch HPLC GmbH) with 62% ACN/20 mM NH_4_OAc_aq_. (pH 6.8) as eluent at a flow of 3.0 mL/min were used. 

The ammonium acetate and the SDS concentrations stated as 20 mM NH_4_OAc aq. and 100 mM aq., respectively, correspond to the concentrations in the aqueous component of an eluent mixture.

#### 4.2.2. Radiosyntheses

No carrier added [^18^F]fluoride (100–500 μL) was transferred into a 4 mL V vial and TBAHCO_3_ (20 μL of a 0.075 M aqueous solution from ABX Advanced Biochemical Compounds, Radeberg, Germany) in 1 mL ACN was added. The aqueous [^18^F]fluoride was azeotropically dried under vacuum and nitrogen flow within 7–10 min using a single mode microwave (75 W, at 50 °C–60 °C, power cycling mode). Two aliquots of anhydrous ACN (2 × 1.0 mL) were added during the drying procedure and the final [^18^F]TBAF was dissolved in 500 μL of anhydrous *tert*-BuOH ready for labelling. Thereafter, a solution of 2.0–2.5 mg of precursor in 300 μL of anhydrous *tert*-BuOH was added, and the ^18^F-labelling was performed under thermal heating at 100 °C for 15 min. To analyse the reaction mixture and to determine labelling efficiencies, samples were taken for radio-HPLC and radio-TLC. Moreover, the stability of the tosylate precursor was investigated under the labelling conditions by using UV-HPLC analysis.

After cooling, 2.5 mL of water and 0.5 mL of ACN were added and the mixture was directly applied to an isocratic semi-preparative RP-HPLC system for isolation of [^18^F]**ICF24027**. The collected radiotracer fraction was diluted with 40 mL of H_2_O to perform final purification by sorption on a preconditioned Sep-Pak**^®^** C18 light cartridge (Waters, Milford, MA, USA) and successive elution with 0.6 mL of ethanol. The solvent was reduced under a gentle argon stream at 70 °C and the desired radiotracer formulated in sterile isotonic saline containing 10% EtOH (*v*/*v*). The identity and radiochemical purity of [^18^F]**ICF24027** was confirmed by radio-HPLC and radio-TLC. Specific activity was determined on the basis of a UV/mass calibration curve carried out under isocratic HPLC conditions (54% ACN/20 mM NH_4_OAc_aq._, pH 6.8) using chromatograms obtained at 254 nm.

#### 4.2.3. *In Vitro* Stability and Calculation of Log*D* Value

The stability of [^18^F]**ICF24027** was investigated by incubation of small tracer amounts of the radiotracer (10–15 MBq) at 40 °C in 1 mL of the following solutions: (i) PBS (137 mM NaCl, 2.7 mM KCl, 10 mM Na_2_HPO_4_; pH = 7.4); and (ii) pig plasma samples. After 60 min aliquots were analysed by radio-HPLC. For the calculation of the log*D* value at pH 7.4, ACD/ChemSketch version 12.5 from Advanced Chemistry Development, Inc. (ACD/Labs, Toronto, ON, Canada), was used.

### 4.3. In Vitro Autoradiographic Studies of *[^18^F]**ICF24027***

Brain sections (20 μm) of flash-frozen brains of female domestic pigs (*Sus s. domestica*, 6 weeks, 12–14 kg) were cut using a cryostat, thaw-mounted onto microscope slides, and after air-drying stored at –80 °C until use. Different mouse tissue sections (12 μm) were obtained by the same procedure from 10 weeks old male CD-1 mice (20–25 g). Briefly, the brain sections were allowed to thaw in air, and rinsed twice in 50 mM TRIS buffer (pH 7.4) to remove endogenous ligand. The sections were then incubated with the 5.26 nM [^18^F]**ICF24027** in TRIS buffer (50 mM TRIS-HCl, 120 mM NaCl, 5 mM KCl, 2 mM MgCl_2_, 2 mM CaCl_2_, 0.1% BSA pH 7.4) for 60 min at room temperature. Nonspecific binding was determined in the presence of 10 μM **ICF24027**.

Displacement of [^18^F]**ICF24027** was also evaluated with 100 nM sildenafil. Subsequently, the sections were washed twice for 2 min in ice-cold TRIS buffer, and rinsed for 5 s in ice-cold distilled H_2_O. The sections were rapidly dried in a stream of cold air before being exposed for 30 min to a tritium-sensitive imaging plate. Developed autoradiographs were analysed in a phosphor imager (BAS-1800 II, Fujifilm). The quantification was performed by using 2D-densitometric analysis (AIDA 2.31 software; raytest Isotopenmessgeräte GmbH).

### 4.4. Metabolism Studies of *[^18^F]**ICF24027*** in Mice

Method (A)—MLC: Blood samples of mouse were taken at 30 min after intravenous injection of ~55 MBq of [^18^F]**ICF24027** (*n* = 2). Plasma was obtained by centrifugation of blood at 12,000 rpm at 4 °C for 10 min. For preparation of the MLC injection samples, mouse plasma (20–50 μL, 30 min p.i.) was dissolved in 100–300 μL of 200 mM aqueous SDS. Homogenized brain material (100–200 μL, 30 min p.i.) was dissolved in 500 μL of 200 mM aqueous SDS, stirred at 75 °C for 5 min and injected into the MLC system after cooling to room temperature. The MLC system was built up of a JASCO PU-980 pump, an AS-2055*Plus* auto injector with a 500 μL sample loop, and a UV-1575 detector coupled with a gamma radioactivity HPLC detector (Gabi Star, raytest Isotopenmessgeräte GmbH). Data analysis was performed with the Galaxie chromatography software (Agilent Technologies). A Reprosil-Pur C18-AQ column (250 mm × 4.6 mm, particle size: 10 μm) coupled with a pre-column of 10 mm length was used. Separations were performed by using an eluent mixture of 1-PrOH/100 mM aqueous SDS/10 mM Na_2_HPO_4_ aq. in gradient mode (0–15 min at 3% 1-PrOH, 15–30 min up to 30% 1-PrOH, 30–45 min at 30% 1-PrOH, 40–50 min up to 3% 1-PrOH; 50–60 min at 3% 1-PrOH/ 100 mM SDS aq., 10 mM Na_2_HPO_4_ aq.) at a flow rate of 0.75 mL/min.

Method (B)—RP-HPLC: Protein precipitation was performed by addition of an ice-cold mixture of ACN/water (4/1; *v*/*v*) in a ratio of 4: 1 of organic solvent to plasma or brain homogenate, respectively. The samples were vortexed for 2 min, equilibrated on ice for 15 min, and centrifuged for 5 min at 10,000 rpm. The precipitates were washed with 200 μL of the solvent mixture and subjected to the same procedure. The combined supernatants (total volume between 1.0–1.5 mL) were concentrated at 65 °C under argon flow to a final volume of approximately 100 μL and analysed by analytical radio-HPLC. To determine the percentage of radioactivity in the supernatants compared to total activity, aliquots of each step as well as the precipitates were quantified by γ counting. To analyse the samples, the same HPLC method was used as described in the radiochemistry part.

### 4.5. Metabolism Studies of *[^18^F]**ICF24027*** with Mouse Liver Microsomes

#### 4.5.1. Chemicals, Reagents and Instruments

Testosterone and NADPH were purchased from Sigma-Aldrich. GIBCO mouse liver microsomes (20 mg/mL) were purchased from Life Technologies (Darmstadt, Germany). PBS Dulbecco buffer (without Ca^2+^, Mg^2+^) was purchased from Biochrom (Berlin, Germany). For conducting microsome experiments the following instruments were used: BioShake iQ (QUANTIFOIL Instruments, Jena, Germany), Centrifuge 5424 (Eppendorf, Hamburg, Germany), Sample Concentrator DB-3D TECHNE (biostep, Jahnsdorf, Germany).

#### 4.5.2. Microsomal Incubations with [^18^F]**ICF24027** and **ICF24027**

All incubations had a final volume of 250 μL and were performed in PBS buffer (pH 7.4) either with 5 MBq of [^18^F]**ICF24027** (specific activity: 107 GBq/μmol) or with a final substrate concentration of 0.2 mM of **ICF24027**. A protein concentration of 2 mg/mL of mouse liver microsomes and 4 mM NADPH were used. Buffer and substrate solutions were mixed with the liver microsomes and preincubated by gentle shaking at 37 °C for 5 min. The reactions were initiated by adding the likewise preincubated NADPH solution to the microsome suspensions followed by gentle shaking at 37 °C. After 120 min the incubations were divided and terminated by addition of cold ACN (−20 °C; ratio of ACN to sample of 4 to 1) for RP-HPLC analysis and aqueous SDS solution (200 mM; ratio of aq. SDS to sample 1 to 1) for MLC analysis, respectively. The MLC samples were directly injected into the MLC system. For the RP-HPLC samples, the mixtures were vortexed for 30 s, stored on ice for 5 min and centrifuged for 10 min (14,000 rpm). The supernatants were separated and concentrated at 50 °C under a nitrogen flow to give residual volumes of 40–70 μL, which were reconditioned by adding of water to obtain sample volumes of 100 μL ready for injection. When [^18^F]**ICF24027** was incubated, two samples of 50 μL each were already taken after 60 min. To one of the samples cold ACN (−20 °C) was added and it was proceeded as described above to obtain an injectable solution for RP-HPLC. The other sample was prepared for MLC by addition of aqueous SDS solution.

Beside the above mentioned batches, incubations either without NADPH or microsomal protein or substrate were analysed as negative controls. For positive control an incubation of testosterone was carried out in similar manner. To analyse the samples, the same MLC and HPLC methods were used as for the *in vivo* studies.
